# 晚期非小细胞肺癌*BRAF*突变靶向治疗进展

**DOI:** 10.3779/j.issn.1009-3419.2018.08.10

**Published:** 2018-08-20

**Authors:** 夏 刘, 殿胜 钟

**Affiliations:** 300052 天津，天津医科大学总医院肿瘤内科 Department of Medical Oncology, Tianjin Medical University General Hospital, Tianjin 300052, China

**Keywords:** 肺肿瘤, 鼠类肉瘤病毒癌基因同源物B1, 靶向治疗, Lung neoplasms, V-raf murine sar-coma viral oncogene homolog B1, Targeted therapy

## Abstract

靶向治疗是驱动基因阳性晚期非小细胞肺癌（non-small cell lung cancer, NSCLC）的重要治疗手段之一。鼠类肉瘤病毒癌基因同源物B1（v-raf murine sar-coma viral oncogene homolog B1, *BRAF*）基因是继表皮生长因子受体（epidermal growth factor receptor, *EGFR*）基因突变、间变性淋巴瘤激酶（anaplastic lymphoma kinase, *ALK*）基因融合和*ROS1*基因重排之后，NSCLC又一个重要的驱动基因。*BRAF* V600E突变占*BRAF*基因突变的一半以上，是晚期NSCLC的潜在治疗靶点，本文主要对*BRAF*基因突变类型及相关靶向研究进展进行综述。。

## Abstract (en)

Targeted therapy is one of the major treatment modalities in advanced non-small cell lung cancer (NSCLC) with sensitive driver gene mutations. *BRAF* is considered a promising oncogenic driver in NSCLC after the discovery of epidermal growth factor receptor (*EGFR*) mutation, anaplastic lymphoma kinase (*ALK*) fusion and *ROS1* rearrangement. *BRAF* V600E mutation accounts for more than half of *BRAF* mutations, which is a potential therapeutic target for advanced NSCLC. This review aims to summarize the advancements of *BRAF* gene mutation and targeted therapy for *BRAF* mutation in NSCLC.

根据《2018国家癌症报告》，肺癌依旧高居中国癌症发病率及死亡率的第一位。非小细胞肺癌（non small cell lung cancer, NSCLC）占全部肺癌的85%左右，初诊时超过60%的患者已失去手术治疗的机会^[1]^。从2004年表皮生长因子受体（epidermal growth factor receptor, *EGFR*）基因突变被发现开始，分子检测技术突飞猛进，越来越多的驱动基因被识别，相应的靶向药物不断涌现，为驱动基因阳性NSCLC患者带来常规化疗无法企及的生存获益，改变了晚期NSCLC的治疗模式^[2]^。本文针对NSCLC中鼠类肉瘤病毒癌基因同源物B1（v-raf murine sar-coma viral oncogene homolog B1, *BRAF*）基因突变及靶向治疗进展作一综述。

## 1 *BRAF*基因结构与突变类型

*BRAF*基因是1988年由Ikawa等^[3]^首先在人类尤文氏肉瘤中发现并克隆确认的，该基因位于染色体7q34，编码丝氨酸/苏氨酸蛋白激酶，与CRAF和ARAF具有较高的同源性，在恶性肿瘤形成、发展过程中发挥重要作用。BRAF蛋白与鼠类肉瘤病毒（kirsten rat sarcoma viral, KRAS）蛋白同为RAS-RAF-丝裂原活化的细胞外信号调节激酶（mitogen-activated extracellular signal-regulated kinase, MEK）-细胞外调节蛋白激酶（extracellular regulated protein kinases, ERK）信号通路中上游调节因子，在丝裂原活化蛋白激酶（mitogen-activated protein kinase, MAPK）/ERK信号通路中起着关键作用^[4]^。RAS-三磷酸鸟苷（guanosinetriphosphate, GTP）与其下游效应分子RAF结合，磷酸化并激活RAF，从而活化RAF下游信号转导MAPK级联途径。RAF蛋白磷酸化并激活下游底物MEK，而MEK磷酸化并激活下游底物ERK，进而调节细胞内的生物学过程^[5, 6]^。

*BRAF*基因突变主要位于CR3激酶结构域的第11外显子及第15外显子，其中*BRAF*最常见突变形式为第15外显子的第1, 799位核苷酸上T突变为A，导致其编码的缬氨酸变为谷氨酸，即V600E，使BRAF蛋白持续激活，提高BRAF活性约500倍^[5]^，激活后的BRAF成为能够不依赖于上游RAS激酶的单体，导致ERK持续激活^[6]^。V600E突变是NSCLC常见的*BRAF*突变类型（55%），其他常见突变包括G469A（35%）和D594G（10%）等^[7, 8]^。与*BRAF* V600突变（V600E/K/D/R）相比，其他类型的*BRAF*突变形成与RAS无关的同源二聚体，不具有单体活性^[6]^。

## 2 *BRAF*基因突变的临床特征

*BRAF*基因突变在人类癌症的发生率为8%，主要是在毛细胞白血病（100%）、黑色素瘤（50%）、甲状腺乳头状癌（45%）等疾病中，NSCLC中突变发生率为1%-4%^[9-11]^。NSCLC的*BRAF*突变的生物学行为和对预后的影响在一些回顾性研究中多次报道。Marchetti等^[8]^报告了1, 046例手术切除NSCLC患者的*BRAF*突变状态，应用聚合酶链反应（polymerase chain reaction, PCR）+高分辨率熔解曲线分析技术（high-resolution melting analysis, HRMA）检测，在总体、肺腺癌和肺鳞癌患者中的*BRAF*突变率分别为3.5%、4.9%和0.3%。其中*BRAF* V600E突变发生率为56.8%，突变患者多为女性、不吸烟、含有侵袭性微乳头成分；非V600E突变者则均有吸烟史。*BRAF* V600E突变患者较野生型患者，无病生存期（disease free survival, DFS）和总生存期（overall survival, OS）明显缩短。Luk等^[12]^针对273例晚期NSCLC患者的*BRAF*、*EGFR*和*KRAS*突变情况进行回顾分析，*BRAF*突变率为2.6%，且均为EGFR及KRAS野生型，突变患者均有吸烟史，性别分布无差异。V600E突变比例58%，与侵袭性微乳头成分相关。而中国肺癌患者的*BRAF*突变率似乎低于欧美，Li等^[13]^采用液相芯片方法回顾5, 125例中国NSCLC患者基因突变情况，*BRAF*突变率为0.5%，且全为V600E突变，多见于女性，但与年龄、吸烟史、组织学类型无关。Ding等^[14]^对1, 680例中国NSCLC患者行扩增阻碍突变系统（amplification refractory mutation system, ARMS）检测，*BRAF*突变比例为1.7%，多见于不吸烟、腺癌患者，其中85.7%为V600E突变，*BRAF*突变型与野生型患者一线化疗的无进展生存期（progression-free sirvival, PFS）无统计学差异。Villaruz^[15]^报道的951例晚期肺腺癌研究显示，*BRAF*突变率为2.1%，多见于吸烟患者，其中81%为V600E突变，比较*BRAF*突变与野生型患者的预后，未发现OS差异。Cui等^[16]^对含有11, 711例NSCLC患者的16项研究进行*meta*分析，*BRAF*总体突变率为2.6%，与腺癌有显著相关性（*P* < 0.000, 1），*BRAF* V600E突变在女性（*P*=0.004）和从不吸烟者（*P* < 0.001）中更常见。上述研究由于人种差异、检查方法不同，结果存在不一致，但总的来说，V600E突变是*BRAF*突变的主要类型，多见于女性、腺癌患者，与预后的关系尚不十分明确，部分研究提示与预后呈负相关。

## 3 *BRAF* V600突变的治疗进展

### 3.1 维罗非尼

维罗非尼作为BRAF特异性抑制剂，2011年被食品药品监督管理局（Food and Drug Administration, FDA）批准治疗具有*BRAF* V600E突变的不可切除及转移性恶性黑色素瘤。Hyman等^[17]^最先报道了维罗非尼在非恶黑恶性肿瘤领域的Ⅱ期研究（VE-BASKET），这也是首个基于组织学分类的篮子研究，在肿瘤治疗领域具有里程碑的意义。全球23个中心共入组*BRAF* V600突变的非恶黑恶性肿瘤（包括NSCLC 20例以及结直肠癌、乳腺癌等7个队列）122例。NSCLC患者应用维罗非尼单药治疗，客观缓解率（objective response rate, ORR）为42%（95%CI: 20%-67%），中位PFS为7.3个月（95%CI: 3.5-10.8），12个月OS为66%（95%CI: 36-85），高于其他类型肿瘤。常见不良反应有皮疹、乏力和关节疼痛（40%）。维罗非尼在这项前瞻性研究中体现出了较好的疗效和安全性，*BRAF* V600突变有望成为NSCLC新的治疗靶点。

研究者随后对NSCLC队列进行扩展，共纳入62例*BRAF* V600突变患者（包括8例初治患者），均接受维罗非尼单药治疗。最终结果在2017年美国临床肿瘤学会（American Society of Clinical Oncology, ASCO）大会报告，经治患者ORR为37.0%（95%CI: 24.3%-51.3%），中位PFS为6.1个月（95%CI: 5.1-8.3），中位OS为15.4个月（95%CI: 8.2-22.6）；初治患者的ORR为37.5%（95%CI: 8.5%-75.5%），中位PFS可达到12.9个月（95%CI: 4.0-NE），中位OS尚未成熟。较经治患者而言，初治患者的PFS显著延长，显示了维罗非尼具有很好的疗效，值得进一步探索。

MyPathway是一个多中心、非随机、多队列的Ⅱa期试验^[18]^，旨在评估适应证以外靶向药物用于含有特定基因变异肿瘤的疗效。入组患者不限瘤种，但必须含有BRAF、HER2、Hedgehog通路等变异，其中*BRAF*突变采用下一代测序（next generation sequencing, NGS）检测，突变患者接受维罗非尼单药治疗。研究共入组251例患者，覆盖35种不同肿瘤类型，包括*BRAF*突变NCSLC患者21例，其中V600突变（均为V600E突变）14例，皆为复发患者。*BRAF* V600E突变患者的疗效与VE-BASKET研究及ACSE研究^[19]^结果较为一致，ORR 43%，中位缓解持续时间（duration of response, DOR）5个月，而非*BRAF* V600突变者的总体人群有效率只有4%。目前，该研究仍在继续，由于*BRAF*非V600突变患者疗效较差，*BRAF*突变队列中将不再入组该类患者。

上述几项研究，证实了篮子试验的潜在价值，维罗非尼用于含*BRAF* V600这一低频突变的NSCLC，显现出令人鼓舞的疗效，后续研究的结果值得期待。

### 3.2 达拉非尼±曲美替尼

达拉非尼作为另一种BRAF抑制剂，是FDA继维罗非尼后批准的第二个治疗伴有*BRAF* V600E突变的转移性黑色素瘤的靶向药物。Planchard教授团队开展了一项达拉非尼单药或联合用于晚期*BRAF* V600E突变NSCLC（BRF 113928）的Ⅱ期试验，结果令人欣喜。研究分为两个阶段。第一阶段对经治*BRAF* V600E突变的NSCLC患者行达拉非尼单药治疗，其中8例评效PR，ORR为40%（8/20），达到主要终点，成功启动第二阶段研究。

在第二阶段，分为以下三个队列进行评估：达拉菲尼单药组（队列A），达拉非尼与曲美替尼联合治疗经治患者组（队列B）及达拉非尼与曲美替尼联合治疗初治患者组（队列C）。

在队列A中，达拉菲尼单药治疗84例*BRAF* V600E突变晚期NSCLC患者（其中78例为经治患者），ORR为33%（95%CI: 23%-45%），DCR为58%（95%CI: 46%-67%），DOR为9.6个月（95%CI: 5.4-15.2）^[20]^。6例一线治疗患者的疗效更为突出，4例获得PR，PFS分别为4.5个月、8.6个月、11.0个月和16.6个月。达拉非尼成为首个在*BRAF*突变的NSCLC前瞻性研究中显示有活性的药物。严重不良反应见于42%的患者，其中皮肤鳞状细胞癌、乏力和基底细胞癌最为常见。

曲美替尼是MEK 1/2可逆性抑制剂，主要通过对MEK蛋白的作用，影响MAPK通路，抑制细胞增殖。在*BRAF* V600E突变或*BRAF* V600K突变的晚期黑色素瘤患者中，针对*BRAF*突变和MEK蛋白的达拉非尼联合曲美替尼的双重MAPK途径抑制治疗，对比达拉非尼单药，ORR分别为69%和53%，PFS分别为11.0个月和8.8个月（*P* < 0.01）^[21]^。体外研究证实，MEK-TKI曲美替尼单药对*BRAF* V600E突变NSCLC细胞系中有效^[22]^。沿用黑色素瘤的成功经验，队列B中，给予经治晚期*BRAF* V600E突变NSCLC患者达拉非尼联合曲美替尼治疗，ORR为67%（95%CI: 53%-79%），中位PFS为9.7个月（95%CI: 6.7-16.6），6个月OS为82%^[23]^。这两药的组合，出现严重不良反应者占比56%，其中发热最为常见（16%），其他包括贫血、精神异常、恶心及皮肤鳞状细胞癌等。基于以上结果，欧盟委员会及美国FDA于2017年4月及6月相继批准达拉非尼联合曲美替尼用于*BRAF* V600突变晚期或转移性NSCLC患者^[24]^。

该联合方案继续用于初治晚期*BRAF* V600E突变NSCLC患者（队列C）^[25]^，经过中位15.9个月随访，ORR为64%（95%CI: 46%-79%），DCR为75%（95%CI: 58%-88%），中位PFS为10.9个月（95%CI: 7.0-16.6），中位OS为24.6个月（95%CI: 12.3-未达到），远远优于传统化疗。3/4级不良反应占比56%，包括发热、呕吐、转氨酶升高、低血压等，与传统化疗相比，耐受性好。在*BRAF* V600E突变转移性NSCLC患者的一线治疗中，达拉非尼联合曲美替尼具有优异的疗效，可管理的安全性，使得*BRAF* V600E成为转移性NSCLC的第四个基因组生物标志物，写入美国国立综合癌症网络（National Comprehensive Cancer Network, NCCN）指南，这是肺癌靶向治疗领域又一重要的里程碑。

2018年5月前后1周内，美国FDA先后批准达拉非尼与曲美替尼联合治疗用于治疗无法手术切除的*BRAF* V600E突变阳性的晚期甲状腺未分化癌，以及用于*BRAF* V600突变的Ⅲ期黑色素瘤患者的术后辅助治疗^[26]^，这一强强组合在肺癌辅助治疗领域是否能够争得一席之地，值得期待。

### 3.3 其他药物

在欧洲EURAF回顾性多中心队列研究中^[27]^，晚期*BRAF*突变肺癌患者绝大多数经历过一线化疗。靶向用药包括维罗非尼、达拉非尼或索拉非尼。在*BRAF*突变中，V600E占83%，ORR为53%，PFS和OS分别为5个月和10.8个月。接受索拉非尼治疗的1例患者达到PR。

## 4 *BRAF*非V600突变的治疗进展

BRAF抑制剂可有效抑制V600突变单体，而在非V600突变的二聚体中，药物只能结合其中一个位点，显著降低对第二位点的亲和力及对下游ERK的抑制作用。因此，从理论上来讲，*BRAF*非V600突变的肿瘤对BFAF抑制剂不敏感，体外实验也验证了这一点^[6]^。在欧洲EURAF队列研究中，6例NSCLC患者为*BRAF*非V600E突变，PFS及OS远远低于V600E突变患者；但是，1例G596V突变患者应用维罗非尼获得了PR。作者认为，位于BRAF激酶的活化区域之内（密码子596-600）突变可能对BRAF抑制剂敏感^[27]^。另外，*BRAF*非V600E突变常常与*RAS*突变同时存在^[28]^，也可能是耐药的原因之一。

在黑色素瘤的临床研究中，*BRAF*非V600E突变者对BRAF抑制剂耐药，但对MEK抑制剂有效。在NSCLC细胞系的研究发现，单药曲美替尼在*BRAF*非V600E突变细胞系可上调AKT信号通路，提示该细胞系对单药MEK抑制剂耐药；当应用曲美替尼联合维罗非尼处理细胞系时，AKT通路没有上调，提示在*BRAF*非V600E突变的细胞系，联合治疗可能优于单药曲美替尼^[29]^。Chen等^[30]^发现，BRAF缺失细胞对BRAF抑制剂维罗非尼耐药但对RAF二聚体抑制剂LY3009120敏感。在BRAF缺失的肿瘤模型中，LY3009120使肿瘤生长退化。对于BRAF缺失或BRAF作为二聚体发挥功能的其他非典型*BRAF*突变的患者来说，LY3009120是一种潜在的治疗选择。目前，尚未检索到MEK抑制剂靶向治疗*BRAF*非V600E突变型NSCLC的临床研究结果。

## 5 BRAF抑制剂耐药机制及耐药后策略

BRAF抑制剂对*BRAF* V600E突变的NSCLC患者疗效确切，但大部分患者不可避免会产生耐药。黑色素瘤中BRAF抑制剂的耐药机制较为明确，原发耐药机制主要包括：*RAC1*突变、*PTEN*缺失、细胞周期蛋白调控紊乱和微环境的变化等。继发耐药机制分为依赖MAPK/ERK通路及不依赖MAPK/ERK通路两种，前者包括NRAS体细胞激活突变，BRAF蛋白激酶的截断、扩增或融合，MEK继发突变激活ERK、BRAF扩增等；后者包括RTK过度表达，PI3K/AKT信号通路活性上调等^[31-33]^。在结直肠癌，尽管5%-8%患者携带*BRAF* V600突变，但对BRFA抑制剂原发耐药，机制可能与EGFR途径激活MAPK信号通路有关。

BRAF抑制剂在NSCLC的耐药机制尚未完全阐明。为探讨*BRAF* V600E突变NSCLC对达拉非尼的继发耐药原因，Kim等^[34]^用MV522 NSCLC细胞（V600E）建立达拉非尼耐药（GSR）细胞株，之后用达拉非尼作用于GSR细胞，发现该细胞EGFR上调，激活EGFR-RAS-CRAF通路，并持续激活ERK1/2，增强EGFR介导的RAS活性，导致BRAF-CRAF二聚体的形成和CRAF的反式激活。研究者发现，ERK1/2的持续激活部分依赖于受体相互作用蛋白激酶-2（receptor interacting protein kinase-2, RIP2）的活性，但不依赖MEK1/2的活性。BRAF和EGFR抑制剂联合应用，可阻断ERK信号的再激活，体外和体内试验中均可增加疗效，可尝试对BRAF继发耐药的NSCLC（V600E）患者联合应用BRAF和EGFR抑制剂治疗。

Abravanel^[35]^报道1例62岁的女性晚期肺腺癌患者，肿瘤组织PD-L1表达90%，采用 > 400个肿瘤相关基因的NGS测序提示，组织及血浆均存在*BRAF* V600E突变。患者一线化疗及二线帕母单抗治疗后，疾病进展，应用达拉非尼联合曲美替尼，靶病灶PR，然而，在21周后，肺部及胸部出现新发病灶。胸壁结节的切除活检证实为转移性肺腺癌，组织和血浆的二次检测显示，除了原始的*BRAF* V600E突变外，还继发了*NRAS q61k*突变，提示显性/克隆性耐受机制。在NSCLC中联合应用BRAF和MEK抑制剂的情况下，耐药是否与克服MAPK通路的抑制有关，比如激活其他RAF家族成员或激活PI3K通路，有待进一步研究。

关于耐药后策略，科学家也进行了一系列探索。Comunanza^[36]^团队发现，在*BRAF*突变小鼠模型，BRAF和VEGF的联合抑制导致肿瘤凋亡、持久的肿瘤反应、肺定植减少并延迟对BRAF抑制剂plx 4720的获得性耐药。Xue等^[33]^发现ERK抑制剂与RAF、MEK抑制剂间歇应用于小鼠肺及黑色素瘤移植瘤模型的协同作用，认为这种给药方式值得耐药患者尝试。Smida等^[37]^毛细血管扩张性共济失调症突变（ataxia telangiectasia-mutation, ATM）的丢失会增强*KRAS*或*BRAF*突变型肺癌细胞对MEK抑制的敏感性，因此，肺癌的ATM突变状态可能成为MEK抑制剂应答的生物标志物。Ross等^[38]^在体外研究中证实，丝氨酸生物合成是肿瘤细胞对BRAF抑制剂耐药的关键因素，同时，也验证了吉西他滨预处理可作为NSCLC细胞系对维罗非尼或达拉非尼耐药的敏化步骤。

## 6 小结

*BRAF*基因突变不仅是EGFR-TKI耐药后的机制之一，亦是原发致癌驱动基因及靶向治疗的重要靶点。BRAF抑制剂及MEK抑制剂在*BRAF* V600E突变NSCLC患者中成效显著，已被写入NCCN指南。但在BRAF抑制剂耐药之后，需要更多相关研究为我们提供治疗策略。未来，随着基因检测技术的飞速发展及分子生物学的不断进步，相信针对*BRAF*基因在NSCLC治疗、预后等方面的研究会有更大进步。
